# Transmission of disorder and etiological information: Effects on health knowledge recollection and health-related cognition

**DOI:** 10.1371/journal.pone.0218703

**Published:** 2019-06-21

**Authors:** Asha Ganesan, Yoshihisa Kashima, John Emmanuel Kiat, Ilan Dar-Nimrod

**Affiliations:** 1 School of Psychology, Faculty of Science, University of Sydney, Camperdown, New South Wales, Australia; 2 School of Psychological Sciences, Faculty of Medicine, Dentistry, and Health Sciences, University of Melbourne, Melbourne, Victoria, Australia; 3 Center for Mind and Brain, University of California, Davis, California, United States of America; 4 The Charles Perkins Centre, University of Sydney, Camperdown, New South Wales, Australia; University of Vienna, AUSTRIA

## Abstract

Biased transmission of health knowledge has far-reaching effects on information reproduction and health-related cognitions. We examined whether transmissions of different types of disorder and etiological information influence recollections of health knowledge and evaluations of patients, by simulating the digital transmission of information. Transmission chains of four non-interacting persons (i.e., four generations) were formed. The first generation read three vignettes describing fictitious patients with one of three disorders (physiological, psychological, culture-bound) uniquely paired with one of three etiologies (genetic, environmental, unknown etiology). Next, they evaluated patients’ well-being, rated desired social distance, and recalled the vignettes. These written recollections replaced the original vignettes for a second-generation of participants, whose recollections were used for the third generation and so on. The framing of disorders affected recollections of etiology, in which culture-bound framings resulted in the poorest recall of etiologies. Participants also perceived the culture-bound disorder as the least serious but desired the most social distance from patients diagnosed with it, when compared to other disorders. The study showed that health information is selectively attended to and reproduced, possibly affected by perceived self-relevance. Faulty recollections and framing of disorders affect health cognitions, potentially instigating biased transmission of disorder- and patient-related narratives.

## Introduction

The gulf between scientific progress and public understanding of science is a long-standing and oft-cited challenge for public health, attributable to the rising complexity of scientific problems and diversity in dissemination sources [1,2]. Although research concerning modern medicine continues to progress rapidly, public understanding of health knowledge has lagged, due, in part, to laypeople’s exposure to unverified health information [3]. For example, during the Ebola outbreak in West Africa, misinformation concerning treatment options (e.g., blood transfusion or plant-based medicines) was relayed via Twitter, intermingled with relevant, accurate information [4]. This example is a part of a broader trend of transmission of false information on social media platforms, which becomes embedded in larger cultural understanding of health [5]. The effects of misinformation about health, in particular, are varied [6] and for the most part, negative [7]. As we have become reliant on the internet and social media for transmission of information, health-related knowledge and norms that change in their transmissions from one individual to another may have lasting effects on people’s health behaviors. As such, it is crucial to understand the cognitive mechanisms through which information about health conditions are remembered and communicated.

Evolutionary theories on cultural transmission can serve as a useful basis for understanding how socially-relevant information is selected for and transmitted across individuals over time to become a part of general cultural understanding. Humans have evolved and developed cognitive and communicative capacities, which preferentially select for useful cultural information [8]. This information is relevant to the survival of both individuals and groups of people [8]. For instance, information that helps people navigate their social environments (e.g., gossip) is often transmitted from one person to the next with more accuracy than information lacking in social-relevancy [9]. More generally, information that tends to help its possessors–be they, individuals or groups–to adapt to their local environment tends to be selected and kept in the population. Consequently, this preferential selection can shape what kind of information is integrated successfully, which contribute to the adaptation of individuals and the groups in which they are embedded [8]. In such situations, observation provides useful cultural information [10]. Children’s observation of adults consistently being cautious, such as avoiding an unknown plant, develops into a norm that decreases the likelihood of them engaging in a costly, and potentially fatal, mistake [10].

As information concerning a group’s environment accumulates over generations of individuals, its application extends to more sophisticated and more specialized forms. For instance, when older women pass on information about toxic foods that younger pregnant women should avoid, the information is incorporated into the culture as a taboo, at times [11]. Thus, cultural transmission of health knowledge provides knowledge that is adaptive for facing challenges in people’s environments. However, the most adaptive knowledge is also usually the knowledge to which individuals allocate their cognitive resources the most [12]. Cultural knowledge may range from elements that are relevant for survival (e.g., avoiding toxic foods) to less dangerous ones (e.g., figuring out the least demanding route home [13]).

When this cultural transmission perspective is applied in the area of health, it helps us understand micro-level psychological processes involved in the transmission of health information [13]. Lay understanding of health conditions tends to be shaped by local culture, in that local folk knowledge and cultural practices often override scientifically-based medical advice in various regions around the world [14,15]. In these situations, beliefs about health or disorder etiologies significantly affect people’s health-related decisions [16–18]. Several studies have documented that a disorder framed as having a genetic cause, in particular, can affect memories and evaluations of both the patients and the disorder itself. For instance, health conditions with genetic etiologies tend to be seen as more immutable or permanent [17], but also patients tend to attract less blame for related actions because the conditions are seen to be outside their control [19]. Likewise, in clinical settings, biological explanations for disorders often make clinicians less empathetic toward patients compared to psychosocial explanations [20]. Generally, empirical studies indicate that both clinicians [21] and ordinary people [22,23] are susceptible to genetic or biological essentialist thinking–where members of a category are perceived to possess an underlying fundamental essence determined by their genetic makeup.

Beyond clinical settings, health-related research is often misunderstood or misapplied by ordinary people, shaping their biases [24], fears, and false beliefs about modern medicine and genetics [25]. Such processes hinder the appropriate use of health knowledge by the general public in making informed health decisions [26], such as in understanding pre-existing conditions and genetic testing needs [27]. Much of these misunderstandings become integrated into the larger cultural sphere to affect both health and social decisions. For example, the threats of illnesses [28,29] and pandemics [30] affect non-diagnosed individuals’ social decisions such as voting behavior and support for social policies as well as increasing desired social distance from diagnosed individuals. Furthermore, the selective transmission of specific health information can lead to biased views about who should get medical or clinical help [31] as well as who should receive high-quality treatment [32].

People acquire biased health knowledge from several sources including familial intergenerational transmission [33] and traditional or digital media transmission [13]. Given the “viral” nature of many online health campaigns, in which ordinary people serve as conduits for such messages to others in their social circle, selective attention to specific aspects of complex health conditions may be enhanced through mediations by non-professionals, leading to a biased understanding of patients’ lives. For example, Angelina Jolie’s op-ed [34] concerning genetic testing for familial breast cancer has been singled out for increasing awareness of a specific type of hereditary breast cancer [35]. However, awareness of such cases does not necessarily improve understanding of the genetic mechanism involved [36] and has resulted in increased requests for genetic testing and intervention, even when there is no familial history of that condition [23]. Thus, it is worth examining whether certain features of a health condition such as its etiology (e.g., whether genetic or not), facilitate its successful and widespread transmission. As the transmission of specific health information (e.g., etiology or disorder) influences people’s health and social decisions, it is important to understand if the changing of information across transmission chains affects people’s views concerning the health condition itself, treatment of participants, and potential interactions with patients.

To test whether genetic essentialist thinking influences decisions in the face of a health-related challenge, we examine the extent of successful transmissions of specific kinds of etiologies (or causal information). We contend that if genetic information influences health decisions, in addition to being recalled more frequently in transmissions, it may also influence people’s views of the health condition, treatment for patients, and their potential interactions with patients compared to other etiological information. Given past studies linking genetic etiology and out-group biases [37], we also consider whether a culture-bound disorder, framed as affecting an outgroup, is recalled less frequently in transmissions and whether patients diagnosed with the disorder are evaluated more negatively.

### The present study

The present study assesses the effects of transmission of disorder and etiological information, through a simulation of digital media transmission. In the study, participants were exposed to vignettes with different etiologies—genetic, environmental, and unknown etiology—paired with disorders (see [37,38])—physiological, psychological, and culture-bound—and asked to evaluate each vignette. Each of the three disorders had unique names, age ranges of typically-affected patients, and symptoms. Participants in the first wave of data collection (i.e., first generation) recorded their recollection of these vignettes. These written recollections were then used by participants in the next wave who in turn recorded their recollections for the subsequent wave and so forth for four waves (i.e., four generations). To our knowledge, the transmission of health knowledge in this manner and its effects on health-related cognition, as a parallel to real-life transmission of information on digital media, has yet to be experimentally examined in a controlled lab setting.

Given the research reviewed above on the transmission of useful cultural information and high salience of disorder etiologies in people’s health and social judgements and decisions, we hypothesized that participants exposed to genetic etiology, compared to other etiologies, will (1) reproduce etiology-related information more consistently across the generations [36], (2) evaluate patient well-being more negatively [22], and (3) will desire more social distance from patients [39,40]. We also expected the least self-relevant health condition—the culture-bound disorder, which was framed as affecting an outgroup [40]—to be recalled the least and devalued the most in evaluations of patients, leading to an increase in desired social distance. Lastly, over generations, participants were expected to make more positive patient well-being evaluations and desire less social distance, due to the dilution of information [9].

## Method

### Participants

Relevant generational learning experiments show effects ranging from small to large (e.g., [36,41]). Considering this range, to detect Cohen’s *d* = 0.50 (a medium effect [42]) for the planned comparisons with at least .80 power, approximately 204 participants were required. After excluding incomplete data from two participants, a total of 198 first-year psychology undergraduates (131 women, 48 men, 19 genders unreported; mean age = 19.16 years, standard deviation = 3.49, range = 18–48 years) participated voluntarily in return for course credit, with an additional incentive of being placed in the running for a $10 voucher. Participants identified as European/White (77), East Asian (33), Mixed ethnicity (27), South Asian (14), Southeast Asian (12), Arab/Middle Eastern (10), and African (4). Others were either unreported (19) or identified as “Other” (2). Participants were mostly native English speakers (143). Participants were born either in Australia (129) or overseas (51), with the remainder opting out of reporting their country of birth (18). Fifty-eight participants were in Generation 1, 62 participants in Generation 2, 45 participants in Generation 3, and 33 participants in Generation 4.

### Measures

#### Vignettes

Three unique vignettes depicting one of three disorders types (i.e., physiological, psychological, or culture-bound) were paired with one of three etiological explanations (i.e., genetic, environmental, or unknown etiology). Each disorder (adapted from [38,39]) had corresponding symptoms such that a physiological disorder was characterized as a physiological (i.e., optic) condition, a psychological disorder was represented by cognitive impairments, and a culturally-bound disorder that occurs exclusively in a particular population (see Table 1). All disorders and purported patients were fictitious. Vignettes were reviewed by five experts and ten undergraduates for open-ended feedback on the clarity of expression as well as the appropriateness of disorder designations, and minor revisions were made to the final versions. Vignette sets were counterbalanced based on an orthogonal Latin square design (see Table 2), which ensured participants only received one set containing three vignettes and that they were exposed to all disorder types and all etiologies only once.

**Table 1:** Example of Disorder-Etiology paired vignettes.

Disorder-Etiology Vignettes		
Physiological-Genetic	Psychological-Unknown Etiology	Culture Bound-Environmental
Leber’s optic disorder is related to **mutations in four different genes found in DNA cellular structures**. The disorder usually appears in 18 to 30 year-olds. The first symptoms of Leber’s optic disorder are the blurring and clouding of vision. Over time, vision in both eyes worsens with a severe loss of visual acuity (sharpness) and colour vision. The condition mainly affects central vision, which is needed in detailed tasks such as reading, driving, and recognising faces.	Johnston-Marcus disorder is a **condition that is relatively unknown and is recommended for further research**. This disorder is characterised by uncontrolled movements, emotional problems and loss of thinking ability. The disorder usually appears in 30 to 40 year-olds. Early symptoms of Johnston-Marcus disorder can include irritability, depression, poor coordination and trouble learning new information. They also experience changes in personality and a decline in thinking and reasoning abilities.	Methinismus is a condition **related to the presence of ANF toxins in the near environment** is exclusive to a small tribe in East Africa. This disorder is characterised by mild obsession with death or a deceased person. This disorder occurs in individuals of 18–85 years old. Other symptoms of Methinismus include weakness, fatigue, and diminished appetite. People with this disorder may experience nightmares, anxiety, and a sense of being in danger.

*Note*: Bolded phrases are etiological information, paired with the corresponding disorder, depending on which vignette set a participant is assigned.

**Table 2:** Vignette sets randomization using an orthogonal Latin square design.

Etiology/Disorder	Genetic	Environmental	Unknown
**Physiological**	Set 1	Set 2	Set 3
**Psychological**	Set 2	Set 3	Set 1
**Cultural-bound**	Set 3	Set 1	Set 2

#### Patient well-being evaluation

Participants rated each patient described in the vignettes on four items (adapted from [21,22]), which were standardized to create a composite patient evaluation measure with higher scores indicating greater well-being (α = .68). All participants were presented identically-worded items for all vignettes. First, using a 7-point scale (1 = *not at all serious*, 7 = *very serious*), participants rated the item “How serious is this disorder to allocate at least 5 million annually to research it?” Then, participants responded to one item on the likelihood of patient obtaining positive health outcomes (1 = *extremely unlikely* to 7 = *extremely likely*; “Based on what you know about this disorder, what are the chances of a person with this disorder living a healthy life?”) and one item on the effectiveness of psychotherapeutic treatment (1 = *not at all effective* to 7 = *very effective*; “How effective do you think psychotherapy would be for this disorder?”). Lastly, using a 6-point scale (1 = *a great deal*, 5 = *none at all*, with an option for “I don’t know”), participants rated one item stating “A patient has been diagnosed with this disorder. To what extent is the person in control of their condition?”

#### Social distance

Participants answered the question, “How comfortable would you be with your sibling dating someone with this condition?” using a 7-point scale (1 = *not at all comfortable*, 7 = *very comfortable*;[43]).

### Procedure

Participants arrived at a lab for a study titled “Investigation of Decisions made about Health Conditions.” They were assigned a computer by an experimenter, who then verbally introduced the study, instructed participants to read through an information sheet and provide written consent (obtained from all participants). The study began with brief instructions informing participants that they will read three vignettes, which they will be asked to recall at a later time. To incentivize accurate recall, they were told the best recollection would receive a $10 gift card. First generation participants were randomly assigned to their vignette set by the software Qualtrics, ensuring that the experimenter remained blind to participants’ experimental condition. They read the first vignette, then completed the evaluation of patient measures. Next, they typed their open-ended recollections in a text box, using their own words.

These steps were repeated twice more for the other two vignettes in the set, with vignette order being randomized for each set. Then, participants completed a final question to identify information from each disorder, after which they completed demographic items (i.e., age, sex, ethnicity, native language, and their university major and minor). At the end of the 25-minute session, participants were verbally debriefed on the true purpose of the experiment, at which time the fictitious nature of the disorders was revealed. Participants in the second generation experienced the same procedures as the first. However, the vignettes presented to participants were the recollections of the participants from the first generation, thus simulating the transmission of the disorder information from the first to the second generation. This transmission chain continued with the second generation’s recollections used for the third generation, and finally, the third generation’s recollections used for the fourth generation. Recollections were not edited, except for minor punctuation corrections. All study procedures were approved by the University of Sydney Human Research Ethics Committee, in line with the Declaration of Helsinki.

### Analysis plan

Statistical analyses were conducted through three separate generalized linear-mixed effects models for the three dependent variables: (1) whether or not etiology was recalled correctly [binomial outcome], (2) evaluations of patient well-being [continuous outcome], and (3) ratings of desired social distance [continuous outcome]. This analytic approach allows assessments of effects at the within-person level (e.g., a participant’s probability of recall of the correct etiology type relative to their probability for mentioning the other incorrect etiology types) and between-person level (e.g., a participant’s likelihood of correct recollections of etiology relative to other participants). Subject-level dependencies were handled by including a random intercept for subjects [44] before entering Etiology Type (Genetic, Environmental, Unknown), Disorder Type (Physiological, Psychological, Culture-bound), and Generation (1, 2, 3, 4) into the model as fixed effects. Effect size estimations for the simple effects were conducted using odds ratio (*OR*) for the binomial outcome and Cohen’s *d* for the two continuous outcomes [45]. All models reported are final models and uncorrected *p*-values are reported. Analyses were performed using PROC GLIMMIX and PROC MIXED in SAS 9.4 for Windows [46], and all data were de-identified prior to analysis. Normality in residual distribution for models was assessed by visual inspection of the Scatter and Quantile-Quantile plots for conditional residuals. No extreme outliers were observed for either continuous outcomes. However, the ratings of desired social distance showed skewed conditional residuals, elaborated further below.

## Results

### Etiological recollection

Correct etiological recollections were identified using a nominal scale: 0 = *Incorrect*; 1 = *Correct* (see Supporting Information for coding methods). Inter-rater reliability for the final coding was assessed using Krippendorff’s alpha and showed appropriate levels at .73 [47]. The model was estimated with all main and two-way interaction terms. This model converged successfully and is reported on below. We also report the mean probability of accurate recollection (*P*_*acc*_) as a part of the descriptive statistics. In this model, the main effect of Etiology Type, *F*(2,360) = 1.19, *p* = .304, on etiological recollection was not statistically significant. However, the main effect of Disorder Type did meet our criterion for statistical significance, *F*(2,360) = 4.40, *p* = .013. Specifically, participants showed higher probabilities of recollecting etiologies paired with the Psychological Disorder (*P*_*acc*_ = .22, *SE* = .04) relative to the Physiological Disorder (*P*_*acc*_ = .12, *SE* = .03), *t*(360) = −2.18, *p* = .030, *OR* = 0.50. Participants also showed higher probabilities of recollecting etiology paired with Psychological Disorder than Culture-bound Disorder (*P*_*acc*_ = .10, *SE* = .03), *t*(360) = 2.63, *p* = .009, *OR* = 2.51. However, their recollections of accurate etiology did not significantly differ between Physiological and Culture-bound Disorder, *t*(360) = 0.56, *p* = .574, *OR* = 1.24.

In this model, the main effect of Generation was also statistically significant, *F*(3,193) = 10.26, *p* < .001. Participants in Generation 1 (*P*_*acc*_ = .36, *SE* = .05)–the generation with access to the complete version of the vignettes–were more likely to recall correct etiological information as compared to participants in all other generations: Generation 2 (*P*_*acc*_ = .12, *SE* = .03), *t*(193) = 4.36, *p* < .001, *OR* = 4.36, Generation 3 (*P*_*acc*_ = .10, *SE* = .03), *t*(193) = 4.02, *p* < .001, *OR* = 4.97, Generation 4 (*P*_*acc*_ = .07, *SE* = .03), *t*(193) = 4.18, *p* < .001, *OR* = 7.17. However, the likelihood of recalling correct etiological information did not significantly vary between the three subsequent generations: Generation 2 and 3, *t*(193) = 0.32, *p* = .748, *OR* = 1.14, Generation 2 and 4, *t*(193) = 1.06, *p* = .291, *OR* = 1.65, and Generation 3 and 4, *t*(193) = 0.71, *p* = .477, *OR* = 1.44.

**Fig 1:**
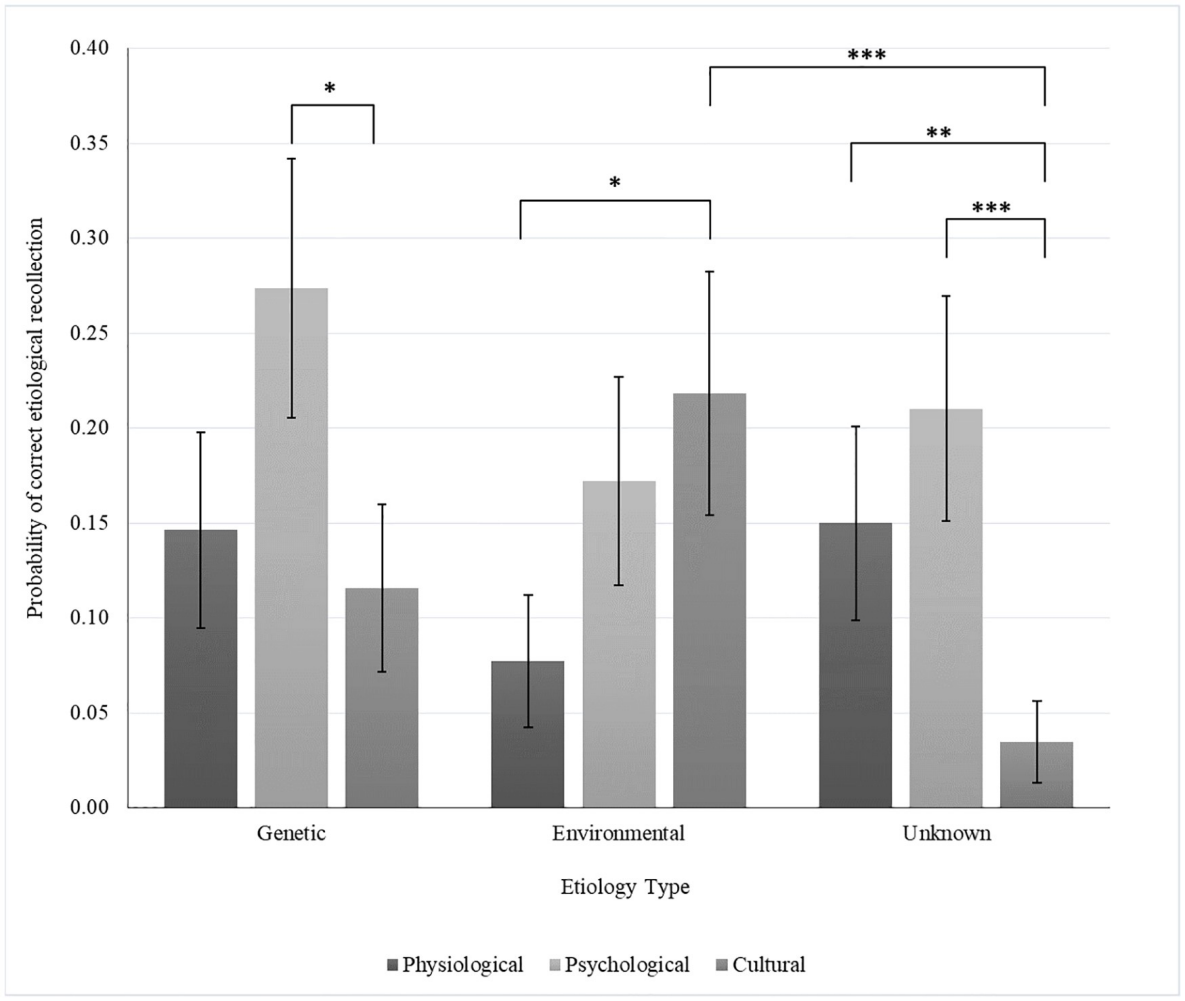
Two-way interaction of Etiology Type and Disorder Type on probability of correct etiological recollections (with SE_mean_). *Note*: *** *p* < .005; ** *p* < .030; * *p* < .050.

The moderating influence of Etiology and Disorder type on the observed generation effect was also not statistically significant (*F*(6,360) = 0.77, *p* = .591, and *F*(6,360) = 0.75, *p* = .611 respectively). The interaction between Etiology Type and Disorder Type with regard to accurate etiological recollection did, however, meet our significance criterion, *F*(4,360) = 2.97, *p* = .020, see Fig 1 for the probabilities of accurate recollection. The simple effects (see Table 3) associated with this interaction indicate that participants were more likely to recall Genetic Etiologies when paired with Psychological Disorders than when paired with Culture-bound ones. Furthermore, Environmental Etiologies were also more likely to be recalled when paired with Physiological relative to Culture-bound Disorders. In the Unknown Etiology condition, participants showed higher recall probabilities for both Physiological and Psychological Disorders compared to Culture-bound Disorder. Finally, a comparison across etiologies (see Table 4) indicates that the only statistically significant simple effect was present for Culture-bound Disorders wherein participants were more likely to recall Environmental Etiology paired with Culture-bound Disorder relative to Unknown ones. All other etiological comparisons did not meet our statistical significance criterion.

**Table 3:** Simple effects table at Etiology Type level for the probability of correct etiological recollections.

Etiology	Disorder	*t*	*p*	*OR*
Genetic	Physiology vs Psychological	−1.54	.125	0.46
	Physiology vs Culture-bound	0.48	.630	1.31
	Psychological vs Culture-bound	2.01	.046	2.88
Environmental	Physiology vs Psychological	−1.57	.116	0.40
	Physiology vs Culture-bound	−2.07	.039	0.30
	Psychological vs Culture-bound	−0.58	.564	0.75
Unknown Etiology	Physiology vs Psychological	−0.82	.412	0.66
	Physiology vs Culture-bound	2.26	.025	4.89
	Psychological vs Culture-bound	2.89	.004	7.39

**Table 4:** Simple effects table at Disorder Type level for the probability of correct etiological recollections.

Disorder	Etiology	*t*	*p*	*OR*
Physiological	Genetic vs Environmental	1.23	.219	2.05
	Genetic vs Unknown Etiology	−0.06	.956	0.97
	Environmental vs Unknown Etiology	−1.30	.196	0.47
Psychological	Genetic vs Environmental	1.20	.231	1.81
	Genetic vs Unknown Etiology	0.73	.468	1.42
	Environmental vs Unknown Etiology	−0.50	.620	0.78
Culture-bound	Genetic vs Environmental	−1.41	.158	0.47
	Genetic vs Unknown Etiology	1.81	.071	3.63
	Environmental vs Unknown Etiology	2.94	.004	7.74

### Evaluations of patient well-being

**Fig 2:**
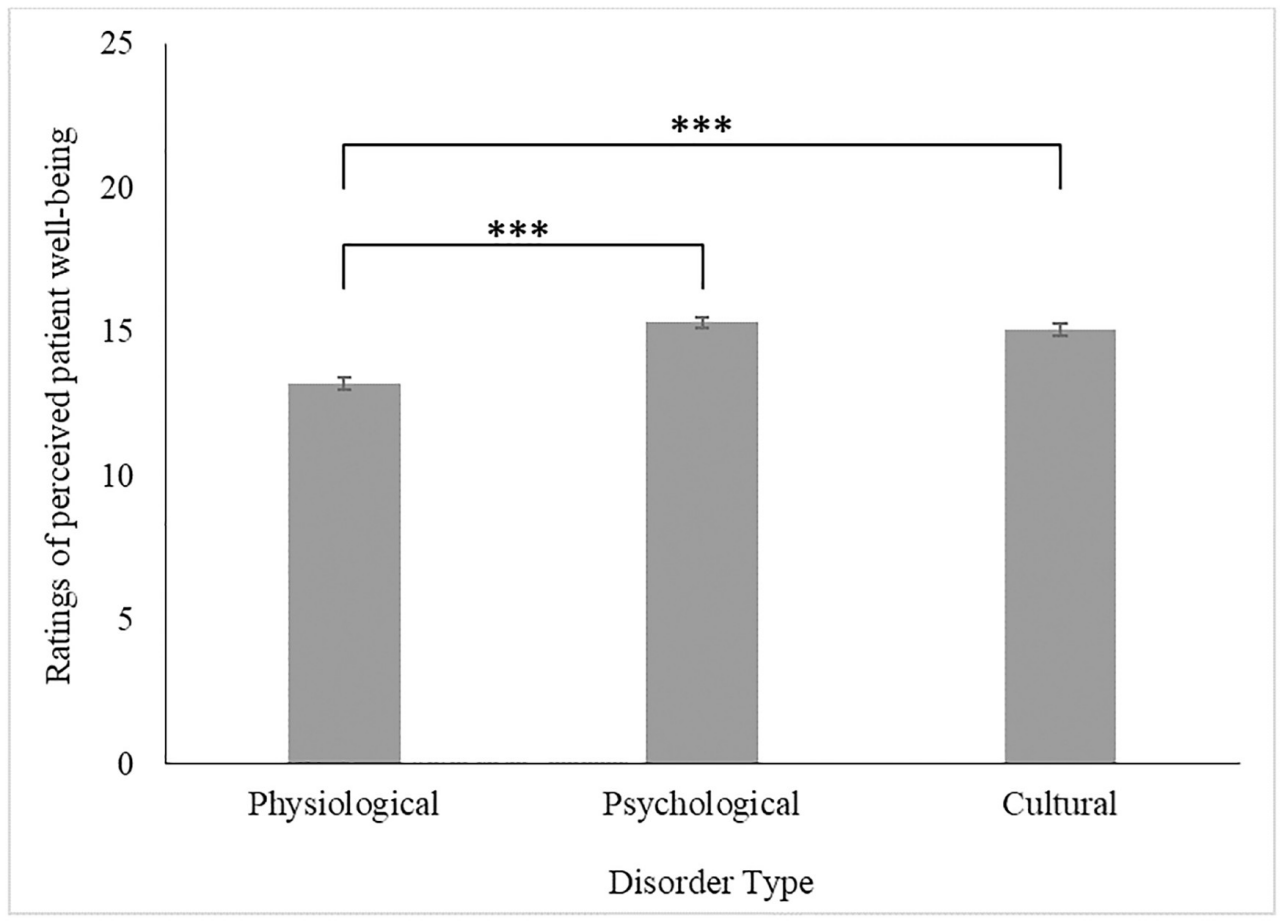
The effect of Disorder Type on ratings of perceived patient well-being (with SE_mean_). *Note*: *** *p* < .005; ** *p* < .030; * *p* < .050. Higher scores are indicative of greater perceived well-being of patient.

As before, the model was estimated with all main and two-way interaction terms. The model converged successfully and is reported below. Higher scores are indicative of more positive well-being evaluation. The main effects of Generation, *F*(3,194) = 0.45, *p* = .719, and Etiology Type, *F*(2,359) = 0.48, *p* = .616, were not significant in this model. The main effect of Disorder Type, however, significantly affected evaluations, *F*(2,359) = 37.84, *p* < .001 (see Fig 2). Contrasts indicated that participants saw the well-being of patients diagnosed with the Physiological Disorder (*M* = 13.21, *SE* = .21) more negatively than those with the Psychological (*M* = 15.32, *SE* = .21), *t*(359) = −7.99, *p* < .001, *d* = 0.84, and the Culture-bound Disorder (*M* = 15.06, *SE* = .21), *t*(359) = −6.99, *p* < .001, *d* = 0.74. Participants’ well-being evaluations did not significantly differ between Psychological and Culture-bound Disorder patients, *t*(359) = 0.99, *p* = .324, *d* = 0.10.

None of the two-way interactions significantly affected evaluations of patient well-being: Generation and Etiology Type, *F*(6,359) = 0.86, *p* = .522, Generation and Disorder Type, *F*(6,359) = 1.86, *p* = .087, and Etiology Type and Disorder Type, *F*(4,359) = 2.05, *p* = .087.

### Desired social distance

**Fig 3:**
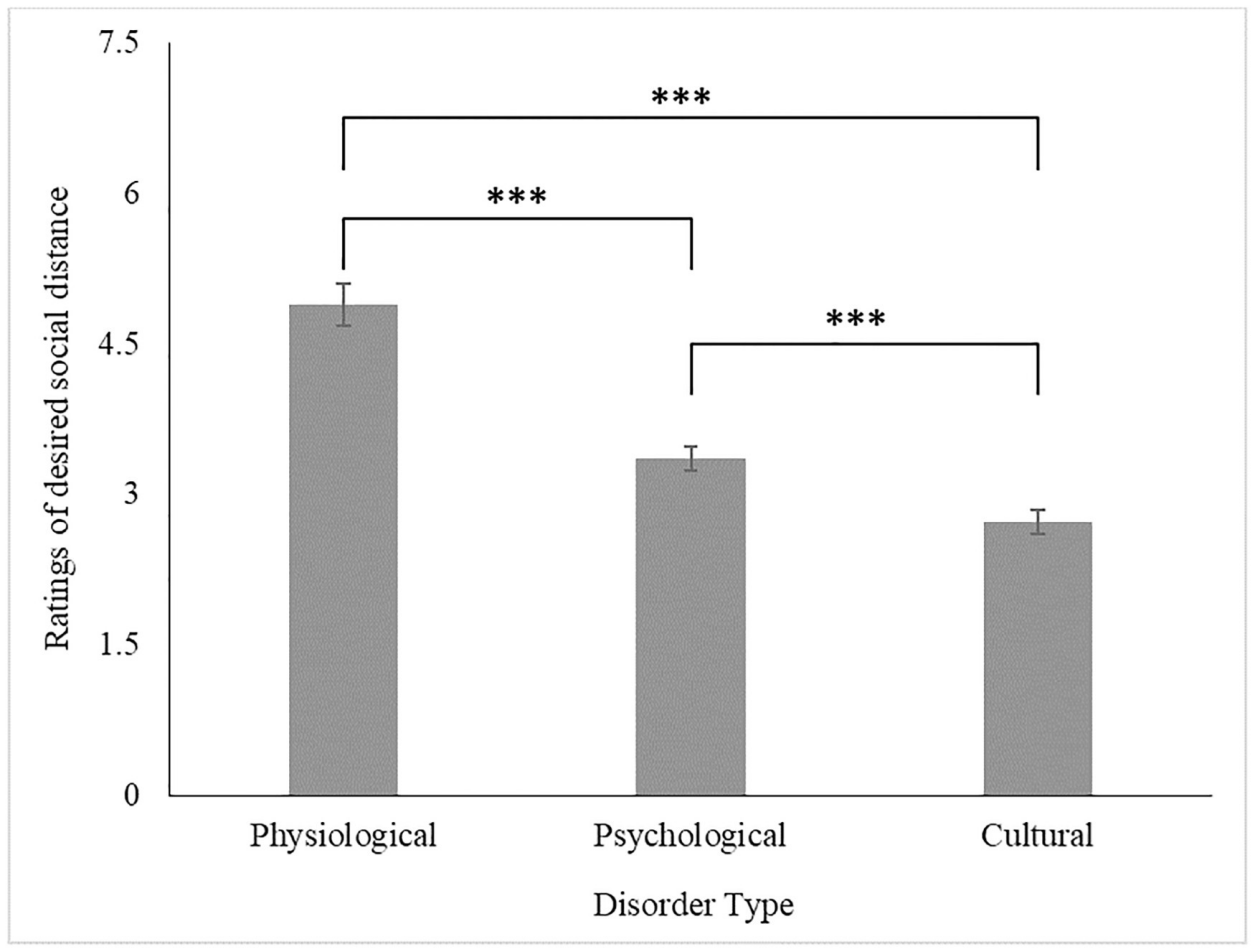
The effect of Disorder Type on ratings of desired social distance (with SE_mean_). *Note*: *** *p* < .005; ** *p* < .030; * *p* < .050. Higher scores are indicative of lower desired social distance.

As with the Etiological recollection model, removal of the three-way interaction proved necessary for successful model convergence. Given the skewed conditional residuals for the outcome, the model was tested using a log-normal distribution. Lower scores are indicative of more desired social distance. The model showed that the main effect of Disorder Type was significant, *F*(2,367) = 106.33, *p* < .001 (see Fig 3). Contrasts showed that participants were most comfortable with their sibling dating a patient diagnosed with the Physiological Disorder (*M* = 4.89, *SE* = .12) compared to the Psychological (*M* = 3.35, *SE* = .12), *t*(367) = 9.12, *p* < .001, *d* = 0.95, and the Culture-bound Disorder (*M* = 2.72, *SE* = .12), *t*(367) = 14.41, *p* < .001, *d* = 1.50. Participants were also more comfortable with their sibling dating a patient with the Psychological Disorder over the Culture-bound one, *t*(367) = 5.30, *p* < .001, *d* = 0.56.

**Fig 4:**
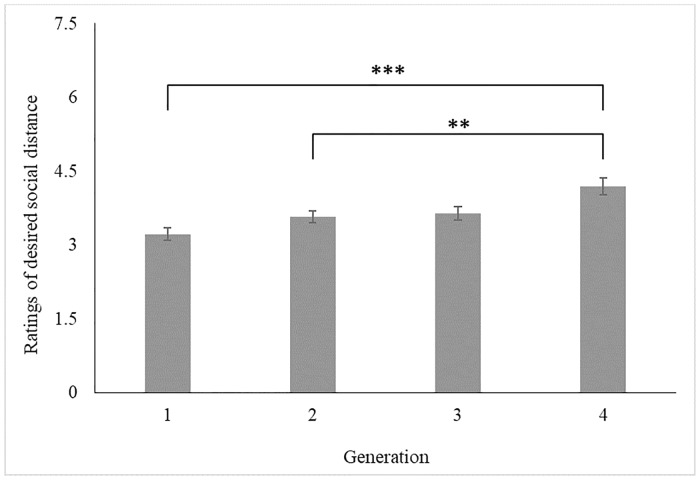
The effect of Generation on ratings of desired social distance (with SE_mean_). *Note*: *** *p* < .005; ** *p* < .030; * *p* < .050. Higher scores are indicative of lower desired social distance.

Desired social distance also significantly varied by Generation, *F*(3,194) = 4.52, *p* = .004 (see Fig 4). Generation 1’s participants (*M* = 3.22, *SE* = .13) desired more social distance from patients than Generation 4’s participants (*M* = 4.19, *SE* = .17), *t*(194) = −3.65, *p* < .001, *d* = 0.52. Similarly, participants in Generation 2 (*M* = 3.57, *SE* = .12) desired more social distance than those in Generation 4, *t*(194) = −2.21, *p* = .028, *d* = 0.32. All other Generations did not significantly affect desired social distance: participants in Generations 1 and 2, *t*(194) = −1.75, *p* = .082, *d* = 0.25, Generations 1 and 3 (*M* = 3.64, *SE* = .14), *t*(194) = −1.93, *p* = .056, *d* = 0.28, Generations 2 and 3, *t*(194) = −0.32, *p* = .747, *d* = 0.05, and Generations 3 and 4, *t*(194) = −1.80, *p* = .074, *d* = 0.26.

The main effect of Etiology Type, *F*(2,367) = 0.38, *p* = .687 did not have a significant effect on social distance. No two-way interactions were significant—Generation and Etiology Type, *F*(6,367) = 1.09, *p* = .366; Generation and Disorder Type, *F*(6,367) = 0.84, *p* = .543; and Etiology Type and Disorder Type, *F*(4,367) = 0.42, *p* = .795.

## Discussion

This study aimed to investigate a few aspects of transmission of health-related information. Drawing on the cultural transmission framework and previous findings concerning disorder etiology, we predicted that: (1) genetic etiology would be more accurately transmitted over generations (i.e., a succession of transmissions) compared to other etiologies, (2) a disorder pertaining to people from other cultures (the least self-relevant) would be devalued as reflected in recollections and evaluation of patients compared to other disorders, and (3) over generations, patients would be evaluated less negatively. Contrary to our hypothesis and prior work [36], participants did not show a significant bias toward genetic etiology in transmission chains. For the most part, etiology did not significantly impact the dependent measures, only doing so for recollections of etiology when combined with the framing of disorders, where the strongest effects were shown for disorders *not* paired with genetic etiology. Substantial differences in the recollection of genetic etiology were only shown when it was paired with a Psychological Disorder compared to a Culture-bound one. Although the transmission and recollection of genetic etiology were both not demonstrated, for the most part, the study adds to the existing evidence concerning biased health cognition [20,21,36] by showing that specific elements of disorders impact the reproduction of health information and evaluations of patients.

One such element concerned whether a disorder was framed as being culturally-bound to an outgroup. In line with the cultural evolutionary framework, etiological information concerning the disorder affecting only an outgroup was recalled and reproduced less than more potentially self-relevant ones, except when the Culture-bound Disorder was paired with Environmental Etiology. These findings may reflect people’s naïve theories about psychological conditions, namely that conditions like schizophrenia or bipolar disorder tend to run in families, but disorders that exclusively affect a cultural group are more likely due to the environment in which the cultural group is situated. They also are consistent with prior work in cultural evolution and intergroup relations, demonstrating that some individuals avoid potential infectious others from their outgroups, potentially to a larger extent than infectious individuals in their ingroups [28,48]. Accordingly, information concerning environmental diseases may take precedence over genetic ones when it is culturally-bound. These biases potentially shape attentional and memory processes, such that social information concerning outgroup members is disfavored unless there is a need to direct attention to them [49]. Though this ingroup bias for social information may provide knowledge that is adaptive to facing environmental challenges, the present study suggests that in the context of health, it manifests in less attention toward genetically-related health information concerning outgroups.

Although the results of the present study did not reveal any generation-based transmission effects for etiology, contrary to most prior research [36,50], the findings highlight the types of social information that may show diverging effects in various contexts. The findings imply that people may be guided not by the genetic information itself but by their intuitions and evaluations of it. What social information people learn varies by culture, age, and context [51], and genetic essentialist thinking as an adaptive response is possibly more salient in certain environments, such as in societies experiencing interethnic conflict [52]. These differences can also create diverging trajectories from a young age, as reflected through cultural differences in evaluation of genetically-based social categories such as race [53]. Thus, the generational transmission of genetic information may be less stable compared to other types of social information, especially when the social context of individuals is also less stable.

Generation did influence participants’ social distance ratings. Earlier generation participants desired more social distance from patients (regardless of disorder and etiology) compared to later generations. However, in this laboratory-based micro-society study, it took up to two generations before social distance ratings significantly changed, parallel to theoretical ideas on the transmission of cultural information, where attitudes or norms do not necessarily change significantly from one generation to the next [54]. Instead, norms or social decisions relevant to one’s environmental challenges are deeply embedded, such that it can take several generations before they show significant and meaningful changes [55]. These embedded norms may also ensure that people learn from or model behaviors of those in their ingroups while creating boundaries for how much they should cooperate with outsiders [54]. The present study suggests that social distance may be one such deeply embedded norm that guides people to set boundaries for social interactions.

Where past randomized experiments have demonstrated the critical role of etiology in evaluating familiar health-related conditions [22,56,57], the current study situates etiology in the context of transmission of cultural information. Past research indicates that genetic attributions lead to less favorable prognosis [20,58], but also lead to increased tolerance and sympathy toward patients [58,59], due to perceived attenuation of patient’s responsibility in having such a health condition [60]. However, other work suggests that the social identities of patients such as their ethnicity or gender, influence the reactions of people, including healthcare professionals, which often manifest in less empathy or more questionable decisions being made for patients who are from the outgroup [31,61]. The present study lends support to this idea, where social evaluations of patients are made through the ingroup-outgroup lens, even when the outgroup is a distant one.

### Limitations and future directions

The current research shows the potential benefit of using both disorder and etiological information in examining the transmission of health information. Unfortunately, we were not able to assess the stability of transmission for the different types of disorders and etiologies, so there is no certainty that the disorder-related effects were not a function of information decay. This is an important limitation of the research, which we attempted to account for with our modeling for various sources of dependencies, but one that needs to be highlighted [62]. Furthermore, one of the limitations in our modeling approach is that responses were treated as independent as opposed to being nested within participants. Our attempt to model the nesting structure failed to obtain reliable estimates, but future studies should investigate this by utilizing more detailed information for specific etiology types in the vignettes.

The study also included a culture-bound disorder to test the effects of etiological information on evaluations of disorders that affect only a specific cultural group; however, the specific culture-bound disorder used may have elicited negative responses by the mere fact of being exclusive to an outgroup population, while no target population was named for the other disorders. Future studies can consider whether information concerning diseases within specific populations (e.g., “exclusive to East Africa”) lead to more negative views of those diseases and afflicted populations, given prior work suggesting that genetic essentialist tendencies influence perceptions of outgroups [63]. Finally, the study used vignettes as primary stimuli, which may be considered artificial, though they were similar to stimuli used in previous research [20,21]. Furthermore, due to the within-subjects design of the present study, the specific details of the vignettes such as patient age group and symptoms varied across conditions. Future work could address this issue with a between-subjects design to better identify the specific types of disorder and etiological information affecting transmission of information and evaluations of patients.

We also contend that with the current design (and designs of prior studies), the effects of new information exposure is limited by the amount of overall information transmitted. Any additional information learned in a brief time needs to be simple enough for more natural assimilation into existing structures [64], while being adaptive and easy to acquire. For example, given the distance between the disorder-susceptible outgroup depicted in the culture-bound disorder (East Africans) and the participants’ location (East Australians), a health threat from a disorder-susceptible outgroup closer to home—one which participants may have more knowledge about—could result in more extreme social distance ratings. Though more than half of our sample identified as ethnic minorities, as undergraduate students, they may be more interested in disorder information presented in this study than individuals in other populations. Further investigation into how existing individual-level knowledge structures impact evaluations of the less self-relevant culture-bound disorder can help in understanding under what conditions health information from an outgroup is less important for one’s own survival needs.

### Conclusions

The study provides initial evidence for the usefulness of integrating macro-level, evolutionary-based notions on intergenerational transmission into studying micro-level, psychological processes through simulations of digital transmissions. The most substantial finding in this study is that the framing of disorders significantly impacted the recollections of etiological information and evaluations of patients. For most parts, participants in this study evaluated disorders independent of their etiology, which is in accord with other works suggesting that specific causal features of the disorder are one of many aspects that a person considers in their health cognition [21,65]. Of the aspects evaluated in the current study, one that seems to influence health knowledge and health cognition consistently is the framing of a disorder as affecting an outgroup.

Thus, the transmission of health knowledge is dynamic and multi-layered, encompassing information on characteristics, causes, and self-relevance. The transmission and transformation of information through social networks allow for many pathways to form, as seen in the transmission of misinformation via social media during critical health epidemics [4,66] and as shown in the results of the present study. Biased selection, encoding, and reproduction of health information create hurdles for fast treatment and recognition of critically-ill patients [31], as well as influence health-related cognitions concerning patients’ diagnoses [36,40]. Further exploration of individual factors influencing the transmission chains and subsequent reproduction of lay health knowledge is necessary for the implementation of measures that ensure ease of understanding, especially since health information is susceptible to changes in networks.

## Supporting information

S1 File Open-ended response coding. (PDF)
