# Supplementary material for: Transmission of disorder and etiological information: Effects on health knowledge recollection and health-related cognition
Source: PLoS One. 2019 Jun 21;14(6):e0218703. doi: 10.1371/journal.pone.0218703 (PMC6588244; doi:10.1371/journal.pone.0218703)
Supplement: S1 File — (PDF) [file pone.0218703.s001.pdf]

Open-ended Response Coding

The open-ended recollections coding was done in stages. First, based on research hypotheses, relevant information was identified for coding - namely etiology, afflicted population description, disorder name, and symptoms. These elements formed the basis for preliminary coding schemes. Then, three trained, independent raters, were assigned the same five sets of recollections, blind to the experimental conditions and generations. The raters coded them with an initial inter-rater reliability value of .69, assessed using Krippendorff's alpha (Krippendorff, 2013), following which raters and one of the authors met to review and reach consensus over discrepant coding. Finally, the raters were randomly assigned recollections for independent coding and any inconsistencies or issues were addressed on a needs-basis with the author. Inter-rater reliability for the final coding was also assessed using Krippendorff's alpha and showed appropriate acceptance levels at .73 (Krippendorf, 2013).

Correct etiology was initially identified using a nominal scale: 0 = *Incorrect*, 1 = *Correct*, and 2 = *Not mentioned.* To ensure consistency in coding, the recollections of any words corresponding to the relevant category were used as correct responses. For instance, for Genetic Etiology (*mutations in four different genes found in DNA cellular structures*), any mention of "*genes*" and/or "*DNA*" were rated as correct. Similarly, for Environmental Etiology (*"the presence of ANF toxins in the near environment"*), recollection of the words "*toxins*" and/or "*environment*" were considered correct responses. For the Unknown Etiology condition, "*a condition that is relatively unknown and is recommended for further research,*" recollection of the keywords "*unknown*" and/or "*research recommended*" were considered correct responses. Close variants of the original keywords were considered in the coding (e.g., *genetic, toxic, don't*

know) based on a list provided by the author. Prior to data analysis, "*Not mentioned*" data were recoded as "*Incorrect*," given that omission of etiology information is an inaccurate recollection, resulting in two levels of coded data, 0 = *Incorrect* and 1 = *Correct*.

References

Krippendorff, K., 2013. *Content analysis: An introduction to its methodology (3rd ed)*. Sage Publications: Thousand Oaks, CA.
